# Clarifying the relationship between coherence and accuracy in probability judgments

**DOI:** 10.1016/j.cognition.2022.105022

**Published:** 2022-06

**Authors:** Jian-Qiao Zhu, Philip W.S. Newall, Joakim Sundh, Nick Chater, Adam N. Sanborn

**Affiliations:** a Department of Psychology, University of Warwick, Coventry, United Kingdom; b School of Health, Medical and Applied Sciences, CQUniversity, Rockhampton, Australia; c Warwick Business School, University of Warwick, Coventry, United Kingdom

**Keywords:** Coherence, Accuracy, Gambling, Rationality, Sampling

## Abstract

Bayesian approaches presuppose that following the coherence conditions of probability theory makes probabilistic judgments more accurate. But other influential theories claim accurate judgments (with high “ecological rationality”) do not need to be coherent. Empirical results support these latter theories, threatening Bayesian models of intelligence; and suggesting, moreover, that “heuristics and biases” research, which focuses on violations of coherence, is largely irrelevant. We carry out a higher-power experiment involving poker probability judgments (and a formally analogous urn task), with groups of poker novices, occasional poker players, and poker experts, finding a positive relationship between coherence and accuracy both between groups and across individuals. Both the positive relationship in our data, and past null results, are captured by a sample-based Bayesian approximation model, where a person's accuracy and coherence both increase with the number of samples drawn. Thus, we reconcile the theoretical link between accuracy and coherence with apparently negative empirical results.

## 1 Introduction

Bayesians, whether in cognitive and brain sciences, artificial intelligence, or statistics, assume that achieving accurate probabilistic beliefs and judgments is assisted by following the coherence constraints of probability theory, as embodied, for example, in graphical probabilistic models. But influential lines of theoretical and empirical research have proposed that, in many real-world situations, coherence is of little value, both in terms of accuracy (Berg, Biele, & Gigerenzer, 2016) and in terms of more general ecological and functional norms (e.g., Gigerenzer, 2019; Hammond, 2000), threatening the foundations of the Bayesian approach.^1^ As a corollary, the probabilistic fallacies exhibited by the heuristics and biases research tradition (Kahneman & Tversky, 1973) would be of little practical consequence (Arkes, Gigerenzer, & Hertwig, 2016).

There are strong theoretical reasons for linking coherence and accuracy. First, perfectly accurate estimates are necessarily also perfectly coherent; any set of probability measures that coheres with reality must necessarily cohere with each other. Second, under reasonable assumptions of accuracy measures, it has been proved that for any incoherent set of estimates there exists at least one coherent set of estimates that is also more accurate with respect to all possible outcomes (Joyce, 1998; Leitgeb & Pettigrew, 2010). This is particularly important, because it means that even when the objective outcome probabilities are not available as a benchmark for accuracy, coherence and accuracy are nonetheless irrevocably related. Thus, even if people have no knowledge about the true probabilities, more coherent estimates are likely to be beneficial in terms of accuracy gains. Although the formal link may be of limited practical value for people estimating the probabilities of complex real-world events, as exactly enforcing coherent beliefs is often computationally intractable (Van Rooij, 2008; van Rooij & Wareham, 2012), coherence is still the benchmark for measuring rationality of human cognition (Tversky & Kahneman, 1974). And if people can at least partially increase their judgment coherence, this implies that there would be a link across individuals as well: those individuals who are better at increasing their coherence would also have increased accuracy.

However, prior empirical studies that have looked at coherence and accuracy between groups or across individuals have not shown evidence of a link. Greater levels of expertise in medical doctors have been shown to increase the accuracy of estimates, but not coherence as measured by extension violations (Reyna & Lloyd, 2006). Professional economists estimating probabilities involving prostate cancer and medical testing displayed near zero correlations between coherence and accuracy (Berg et al., 2016). The one study we have found that indicates some evidence of a link showed that self-assessed snooker experts make more coherent and more accurate probability estimates of tournament match winners, although not on all measures of coherence (Wright, Rowe, Bolger, & Gammack, 1994). In addition, this result was confounded by constraints arising directly from probability theory, because, unlike in the other two studies, the coherence and accuracy measures were derived from the same set of judgments.

Any potential link between coherence and accuracy is likely to be affected by psychological factors such as expertise, motivation, and cognitive capacity. Expertise can qualitatively change the way in which people solve problems and even perceive problems in a domain (Harnad, 1987; Shiffrin & Schneider, 1977), and individuals vary greatly in their cognitive capacities and levels of motivation (Stanovich & West, 2000). If coherence and accuracy are closely linked, as Bayesians typically assume, then increases in expertise, motivation, and/or cognitive capacity (hence enhanced accuracy) should also imply greater coherence. But if there is no link between coherence and accuracy, then we should expect that improvements in these psychological factors, and resulting practical success, does not make people conform any better to the benchmark of Bayesian rationality. Previously cited studies appear to favour this latter perspective, since no clear relationship was observed despite the relatively high statistical expertise of the participants (e.g., medical doctors, professional economists).

Given the clear theoretical link between coherence and accuracy, it is surprising that it has not been demonstrated empirically. In this paper, we reconcile this apparent contradiction. First, we present a high-power experimental design demonstrating a positive relationship between coherence and accuracy that does not seem to depend on expertise. Second, we use an approximation to Bayesian rationality to model this relationship: the Bayesian sampling model of probability judgment (Zhu, Sanborn, & Chater, 2020), using individual differences in the number of samples drawn reflecting individual differences in cognitive capacity. Third, we show that this model can explain the lack of a clear relationship in previous studies, because the expected strength of the relationship depends on the probabilistic set-up. Indeed, a power analysis using the sampling model shows that, in some prior experimental contexts, the link between accuracy and coherence, while positive, would likely be undetectable. Thus, we claim that, in line with theoretical predictions, coherence and accuracy are always positively related (as Bayesians argue) – but there are circumstances in which this relationship is relatively weak (as researchers focussing on accuracy point out) and therefore difficult to detect.

Our experiment focuses on a simple, but high-power paradigm case where true probabilities are objective and fixed, and where some participants have expertise through many years of experience, often with significant financial incentives. We used a key scenario in the most popular form of poker; in Texas hold'em poker, the “flop” is three community (i.e., publicly visible) cards dealt face up that all players use in combination with their private cards, for example, A♥ K♥ K♣. There are three main features which determine how a given flop improves the strength of a player's private cards. The number of identical suit cards used for forming a “flush” can be equal to three, two, or one (here two – the hearts), so can the number of cards in a row capable of forming a “straight” (here also two – ace, king), and so also can the number of cards of equal rank capable of forming three-of-a-kind or better (the two kings). Just after the flop is shown, a player privately holding two more kings has the best possible hand, but a player holding Q♥ J♥ could draw a winning royal flush if the 10♥ appears on one of the two final rounds of the hand. Critical for our study, Texas hold'em involves two more community cards after the flop and players' private cards. Thus, knowing the probability of certain three-card combinations on the flop is an important aspect of becoming a good poker player and expert players will have seen a very large number of flops during their careers, although they are unlikely to have memorized the true probabilities (there are 22,100 unique flops in hold'em poker, 1755 of which are strategically different; Newall, 2013). In order to explore the role of presentation format, we also included questions for a ball and urn task with probabilities matched to those of the poker questions.

**Fig. 1:**
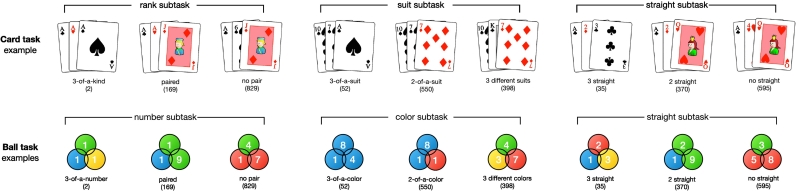
Illustrations of the Card and Ball tasks. Participants estimated the frequency (out of 1000) of outcomes that matched each description, with an example pictured for each (see Appendix B for detailed descriptions of questions). Correct estimates are given in brackets underneath each description. The descriptions in each subtask are mutually exclusive and exhaustive, so the sum of frequencies within each subtask equals 1000.

As shown in Fig. 1, we asked participants about all of these probabilities in the form of frequencies out of 1000. We queried people about mutually exclusive and exhaustive events, where there is a simple and natural measure of coherence: the extent to which these probabilities sum to one. In past work, sum of these probability estimates has been shown to reliably exceed one, an effect termed subadditivity (Adam & Reyna, 2005; Redelmeier, Koehler, Liberman, & Tversky, 1995; Reyna & Lloyd, 2006; Tversky & Fox, 1995). There are of course other measures of incoherence, and while our study was not designed to do so, we can explore the extent of incoherence on these measures to add robustness to our findings. In particular, our stimuli allowed us to look at the number of extension errors (Tversky & Kahneman, 1983) and the degree of inconsistency between matching questions. Extension errors concern about how often participants estimate the probability of a general set to be less than a more restricted set (e.g., judging the probability of 3-of-a-kind, which is three cards with the same rank and hereby must be three cards of different suits in the standard deck of 52 cards, to be higher than the probability of 3-different-suits, which also includes sets of cards that are not 3-of-a-kind). Moreover, the unique design of matching questions between the Card and Ball tasks (see also Fig. 1) enables an investigation on how robust probability judgments were against structurally the same, but differently framed, questions.

We tested whether there is a positive relationship between the coherence and accuracy in two experiments. In the first we recruited participants from the Prolific participant pool, dividing them into poker novices (*N* *=* *45*) and poker amateurs (*N* *=* *37*) depending on whether they reported experience playing poker. In the second experiment, we recruited poker experts (*N* *=* *186*) from the main social community for online professional players (twoplustwo.com), including at least one top professional.^2^

## 2 Method

### 2.1 Participants: Experiment 1

We recruited 139 participants through the Prolific participant pool. Participants were asked to complete a 20-min online survey for a monetary reward (£4) and performance feedback. Performance scores were calculated based on participants' probability estimates and were provided at the end of survey. As stated in our preregistration,^3^ we only included participants who completed the survey and understood the task structure correctly. Before the Card task, participants were asked about the total number of suits and total number of cards per suit in a standard deck of cards. Similarly, before the Ball task, participants were also asked about the total number of colours and total number of balls per colour in the deck-of-cards-equivalent urn. For subsequent analyses, we do not consider participants who provided incorrect responses to any of these queries or failed to complete both Card and Ball tasks. A total of 82 participants (32 female, 49 male, and 1 nondisclosed gender, aged between 19 and 68) remained: 45 of them self-identifying as having no prior poker experience (poker novices) and 37 self-identifying as having some poker experience (poker amateurs). Overall, 82% of poker novices reported playing no poker in the past 12 months, whereas this number drops to 46% for poker amateurs. We asked participants to complete the Gambling Fallacies Measure (GFM), a scale that measures understanding of gambling-specific statistical concepts (Williams, 2003; also see Appendix B). Both poker novices and amateurs scored a median of 6 out of 10 questions correct with no significant differences between the two group (*Welch's t(77.53)* *=* *0.61, p* *=* *0.54, BF*_*10*_ *=* *0.27*).^4^

### 2.2 Participants: Experiment 2

We made a public link to the study available in the main social community for online professional players (twoplustwo.com) for 14 days. A total of 906 respondents were recorded over the 14-day recruitment period, of which 306 respondents completed the experiment. This experiment was the same as Experiment 1 with an additional question about whether they have taken this survey to discourage repeat participation, and the incentives were also different. Because small monetary rewards would not be motivating for professional poker players, participants received aggregate accuracy and coherence scores upon completion of the experiment. In addition, participants were also told their relative ranks in terms of accuracy and coherence compared to the pooled participants in Experiment 1. As preregistered,^5^ the same inclusion criteria were applied to this sample and 186 participants (5 female, 174 male, and 7 nondisclosed gender, aged between 20 and 80) are considered in our subsequent analyses as poker experts. Overall, 66.67% of poker experts reported playing poker on an almost daily basis, while only 3.7% had not played poker in the last 12 months. Poker experts also correctly answered a median of 8 out of 10 questions on the GFM, significantly more than either poker novices or amateurs (with novices and amateurs pooled: *Welch's t(106.35)* *=* *10.28, p* *<* *0.01, BF*_*10*_ *>* *100*).

### 2.3 Procedure

Participants were instructed to estimate the frequencies of different 3-card combinations (i.e., the flop in Texas hold'em) from a standard deck of 52 playing cards. Participants estimated the number of occurrences within 1000 repetitions, a format that has been shown to improve probability estimates compared to eliciting probabilities between zero and one (Gigerenzer & Hoffrage, 1995). To discourage explicit calculation or searching the internet for the answer, participants were given a maximum of 60 s to respond to each question.

To create a set of novel tasks for all participants, they were also asked to estimate the frequencies of 3-ball combinations from an urn of 52 balls which was formally identical to the 52-card deck (see Fig. 1 and Appendix B for details). The urn contained balls of 4 colours (i.e., matching the 4 suits of poker) and a “magic ball” of each colour that can be either the lowest or the highest ranked ball (i.e., matching the Ace).

Participants were first given instructions and tested on the numbers of suits/colours or cards-per-suit/balls-per-colour and then completed nine questions in each task. A representation of the pack of cards or balls in the urn was always displayed alongside the questions. The order of the Card and Ball tasks was counterbalanced across participants, and question order was randomized within each task for each participant. Following the Card and Ball tasks, participants were asked to report how often they played poker in the last year (Recent Poker Experiences), to complete the GFM, and to judge whether they noticed differences between the two tasks (see Appendix F for details).

## 3 Results

**Fig. 2:**
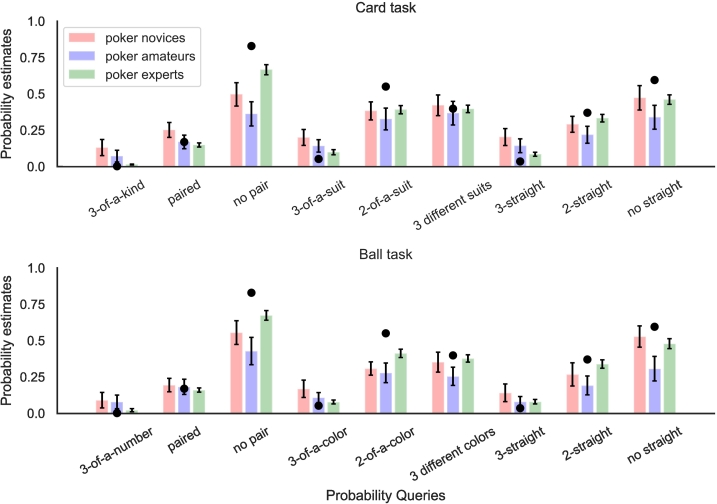
Mean judgments rescaled from frequencies to probabilities for the Card task (top) and Ball task (bottom). Poker novices (red/left bars) and amateurs (blue/middle bars) are the participants from Experiment 1. Poker experts (green/right bars) are the participants from Experiment 2. The black dots are the correct probability estimates. The error bars are 95% confidence interval across participants. (For interpretation of the references to colour in this figure legend, the reader is referred to the web version of this article.)

**Fig. 3:**
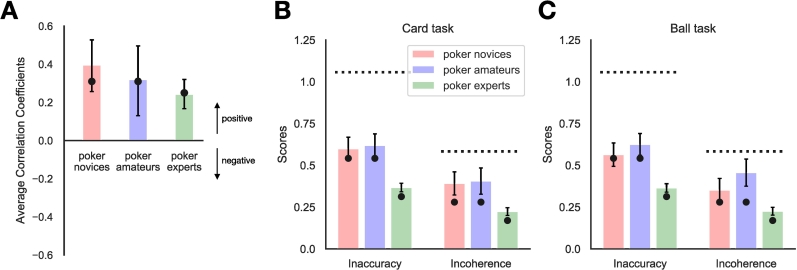
(**A**) The average correlation coefficients between accuracy and coherence. The inaccuracy and incoherence scores of poker novices (red/left bars), amateurs (blue/middle bars), and experts (green/right bars) in the Card task (**B**) and the Ball task (**C**). Detailed correlation results are also shown in Appendix C and D. The dashed horizontal lines represent chance performance levels. The simulation of a Bayesian sampler model in the inaccuracy and incoherence scores and its average correlation coefficients between accuracy and coherence are overlaid as black dots. Error bars are bootstrapped 95% confidence intervals across participants. (For interpretation of the references to colour in this figure legend, the reader is referred to the web version of this article.)

As shown in Fig. 1, participants were asked three mutually exclusive and exhaustive queries within each subtask. The mean estimates, transformed into probabilities, of poker novices, amateurs, and experts are shown in Fig. 2. We can see here that participants overestimated the smallest probabilities and underestimated the largest probabilities. Their incoherence scores for subtasks are the absolute difference between 1 and the sum of the three probability estimates, which can also be thought as maximum sure loss for an expected-value-maximizing gambler on every pound bet (see Appendix A). The inaccuracy scores for a subtask are the sum of three absolute differences between the true frequencies and estimated frequencies. There were no significant differences between poker novices and amateurs in incoherence (*Welch's t(75.95)* *=* *−1.19, p* *=* *0.24, BF*_*10*_ *=* *0.33*) or inaccuracy (*Welch's t(79.71)* *=* *−0.89, p* *=* *0.37, BF*_*10*_ *=* *0.43*) (see also Fig. 3A and 3B). Poker experts, however, made significantly better probability estimates than the pooled novices and amateurs from Experiment 1 in terms of both incoherence (*Welch's t(109.25)* *=* *−6.53, p* *<* *0.01, BF*_*10*_ *>* *100*) and inaccuracy (*Welch's t(124.49)* *=* *−9.11, p* *<* *0.01, BF*_*10*_ *>* *100*).

We investigated whether there were differences between the Card and Ball tasks in either coherence or accuracy. Between the two tasks, there was no evidence of differences in coherence scores for novices (*t*(44) = 1.35, *p* = 0.18, *BF*_*10*_ = 0.38), amateurs (*t*(36) = −1.41, *p* = 0.17, *BF*_*10*_ = 0.44), or experts (*t*(185) = −0.16, *p* = 0.88, *BF*_*10*_ = 0.08). There were also no evidence of differences in accuracy scores for novices (*t*(44) = 1.21, *p* = 0.23, *BF*_*10*_ = 0.32), amateurs (*t*(36) = −0.21, *p* = 0.84, *BF*_*10*_ = 0.18), and experts (*t*(185) = 0.21, *p* = 0.84, *BF*_*10*_ = 0.08). This may have been because many participants (82% of novices, 70% of amateurs, and 93% of experts) noticed that the two tasks were identical or very similar. But even when we restrict the analysis to the small subset of participants (*N* *=* *32* pooled across the three expertise groups) who reported that the Card and Ball tasks were different, we still found no evidence for differences in coherence (*t*(31) = −1.14, *p* = 0.26, *BF*_*10*_ = 0.34) or accuracy (*t*(31) = 0.76, *p* = 0.46, *BF*_*10*_ = 0.25) across the two tasks.

We tested the correlation between our coherence and accuracy scores carefully to avoid confounds. Measuring the coherence and accuracy of the same set of judgments can induce spurious correlations from noisy responding alone, because perfectly accurate estimates are also, by the laws of probability, also perfectly coherent. We therefore only considered the correlations between coherence and accuracy calculated from different subtasks (e.g., coherence of the Card-rank subtask and accuracy of the Ball-colour subtask), so that the separate judgments were used for the two measures. This yielded 30 unconfounded correlations within each group. We found positive correlations across subtasks and for all three expertise groups. Of the 30 unconfounded correlations in each group, 25 were significantly positive (significance level: *p* < 0.05) for novices, 16 were significantly positive for amateurs, and 27 were significantly positive for experts (see Appendix C). None were significantly negative for any of the three groups.

To summarize the magnitudes of the positive correlations between coherence and accuracy, we calculated an average correlation coefficient for each group of participants and bootstrapped 95% confidence intervals as the 30 correlations were not independent. This was done by sampling 10^5^ sets of participants with replacement, calculating the mean correlation coefficients between coherence and accuracy for each set to produce a distribution of mean correlation coefficients, and then choosing the 2.5th and 97.5th percentiles of that distribution. We found significantly positive average correlations between coherence and accuracy for poker novices, amateurs, and experts (see Fig. 3C). However, the mean correlation coefficients of poker novices, amateurs, and experts were not significantly different from one another (bootstrapped differences in mean correlation coefficients between novices and amateurs: 0.0759, 95%CI, [−0.1498–0.3058], between amateurs and experts: 0.0770, 95%CI [−0.1264–0.2708], between novices and experts: 0.1530, 95%CI [−0.0064–0.3056]).

The correlations between coherence and accuracy were not driven by the differences in Recent Poker Experiences (RPE) or tendency to fall into classic gambling fallacies (as measured by the Gambling Fallacies Measure or GFM, see below; Williams, 2003) between the poker experts and the less-expert groups. For each correlation between coherence and accuracy for a pair of subtasks, we calculated partial correlation coefficients that removed the effects of RPE or GFM. We calculated the average partial correlation coefficient over pairs of different subtasks (see Appendix F) and bootstrapped a distribution of differences between the full correlation coefficient and the partial correlation coefficient. These reductions in the correlation coefficients show how much of the correlation between coherence and accuracy was explained by RPE or GFM. For RPE the reductions were −0.0010 (95% CI, −0.0173 — 0.0226) for the novices, −0.0006 (95% CI, −0.0258 — 0.0336) for the amateurs, and −0.0002 (95% CI, −0.0063 — 0.0120) for the experts. For GFM, the reductions were 0.0183 (95% CI, −0.0171 — 0.0904) for the novices, 0.0322 (95% CI, −0.0307 — 0.1472) for the amateurs, and 0.0073 (95% CI, −0.0039 — 0.0252) for the experts. Overall, the reductions in correlation were small and non-significant for both RPE and GFM for every expertise group.

Of course, there are many alternative ways to measure incoherence, so for generality and to complement our main pre-planned incoherence measure we performed an exploratory analysis of extension errors (see Appendix D) and inconsistency between matching questions (see Appendix E). An extension error has been committed when, for any event A that includes event B, but P(B) is judged higher than P(A) (Tversky & Kahneman, 1983). Among our stimuli, there were four possible pairs of judgments within each task that could demonstrate extension errors (e.g., judging the probability of 3-of-a-kind, which is three cards with the same rank and hereby must be three cards of different suits in the standard deck of 52 cards, to be higher than the probability of 3-different-suits, which also includes sets of cards that are not 3-of-a-kind) and we simply counted the number of errors for each participant within each task. We found that poker novices committed significantly fewer extension errors than amateurs, but the evidence is weak (*Welch's t(58.43)* *=* *−2.13, p* *=* *0.04, BF*_*10*_ *=* *1.61*). Poker experts made substantially fewer extension errors than novices (*Welch's t(49.08)* *=* *−2.86, p* *<* *0.01, BF*_*10*_ *=* *7.27*) or amateurs (*Welch's t(37.35)* *=* *−4.10, p* *<* *0.01, BF*_*10*_ *>* *100)*. As the extension error measure never included probability estimates of the “middle” stimulus in each subtask in Fig. 1, we used the total absolute deviation of these judgments from the correct values as our accuracy measure to avoid confounds. We then correlated the coherence of Card and Ball judgments with the accuracy of Card and Ball judgments to produce four correlations for each expertise. We found that the average correlation coefficient (with bootstrapped CIs) was 0.14 (95% CI, −0.04 — 0.32) for novices, 0.18 (95% CI, −0.14 — 0.49) for amateurs, and 0.24 (95% CI, 0.14 — 0.33) for experts. For the poker experts, coefficient was positive and significantly different from zero.

Furthermore, each probability question in the Ball task has a matching equivalence in the Card task (vice versa); and both questions have common underlying combinatorial structure and same solution. Therefore, absolute differences in probability estimates between matching questions can be considered as an alternative incoherence measure. Potentially misinterpreting the Card and Ball task, however, may confound such an incoherence measure. To partially address this concern, the following analyses only include those participants who did not report that the Card and Ball tasks were different in important ways. This results in a sample size of 37, 26, and 173 for poker novices, amateurs, and experts respectively. There is substantial evidence in favour of no difference in inconsistency between poker novices and poker amateurs (*Welch's t(58.03)* *=* *−0.29, p* *=* *0.77, BF*_*10*_ *=* *0.27*). Poker experts, however, were significantly more consistent than novices (*Welch's t(43.76)* *=* *2.88, p* *<* *0.01, BF*_*10*_ *=* *7.88*) and amateurs (*Welch's t(29.96)* *=* *3.13, p* *<* *0.01, BF*_*10*_ *=* *16.17*). As the inconsistency can be computed based on one subtask, we used probability estimates from the other subtasks to form an accuracy measure; avoiding the confound raised from using the same set of judgments to construct both coherence and accuracy measures. We found that the average correlation coefficient between inconsistency and inaccuracy (with bootstrapped CIs) was 0.28 (95% CI, −0.02–0.38) for novices, 0.06 (95% CI, −0.21–0.29) for amateurs, and 0.37 (95% CI, 0.26–0.57) for experts. Detailed correlation analyses can be found in Appendix E.

### 3.1 The Bayesian sampler: modelling the link between coherence and accuracy

We use a recent model of probability estimates, the Bayesian sampler model (Zhu et al., 2020), to reconcile the positive relationship between coherence and accuracy observed above with past work that has generally not found any evidence of a relationship. While doing so we do not claim to show that this model is necessary to match these results, only that it is sufficient to do so, thereby demonstrating that the null-results in previous studies does not conclusively disprove a relationship between coherence and accuracy. This model assumes that participants draw a limited number of samples, *N,* using the true underlying probabilities, either from past experience or mental simulation. However, instead of simply calculating the relative frequency of occurrence to make an estimate (e.g., the proportion of 3-of-a-kind in the set of samples), the Bayesian sampler's estimate is moderated toward 0.5 for small numbers of samples, which explains the overestimation of small probabilities and underestimation of large probabilities that we see in our data. This moderation is motivated by a prior belief that a wide range of true probabilities are likely (captured by a Beta prior distribution), and the model normatively combines this prior belief with the evidence provided by the mental samples to make an estimate. More formally, the Bayesian sampler's estimate is $\frac{M+\beta }{N+2\beta }$ where *N* denotes the total number of mental samples and of which *M* samples were flagged as “true”. For simplicity we assumed that *β* = 1 in all simulations (i.e., a uniform prior).

With an infinite number of random samples, estimates will be both perfectly coherent and perfectly accurate, but as the number of random samples decreases coherence and accuracy will also both decrease. Thus, we need to assume that different participants draw different size samples in order to produce a correlation between coherence and accuracy, and this is congruent with past work showing individual differences in sample size (Zhu et al., 2020). For simplicity, we assumed that participants would draw either a small or large number of samples: *N* *=* *1* or *N* *=* *10*. To qualitatively match the experimental results, we assumed that the populations of poker novices and poker amateurs were 70% *N* *=* *1* and 30% *N* *=* *10*, while the population of poker experts was 10% *N* *=* *1* and 90% *N* *=* *10*. The higher average sample size for experts is consistent with results showing that they consider a greater number of possibilities when playing poker (St. Germain & Tenenbaum, 2011). As shown in Fig. 3, this sampling model reproduces the key empirical results: improvements in incoherence and inaccuracy scores for poker experts, and positive correlations between coherence and accuracy within each group.

We then applied our model to the past results of Berg et al. (2016) and Wright et al. (1994), excluding only the Reyna and Lloyd (2006) study because it did not provide enough information to determine the true probabilities. We modelled the experiment of Berg et al. (2016) using the same Bayesian sampling model and parameters used for the poker experts. Berg et al. (2016) asked 125 professional economists to judge risks associated with prostate cancer. They only asked participants to estimate four probabilities: (1) the probability of getting prostate cancer over the lifetime *P(C*_*lifetime*_*)*, (2) the probability of death from prostate cancer over the lifetime *P(D*_*lifetime*_*)*, (3) the probability of prostate cancer given a positive test *P(C|+)*, and (4) the probability of a positive test given prostate cancer *P(+|C)*. Based on medical literature, the true values of probabilities were *P(C*_*lifetime*_*)* *=* 0.177 and *P(D*_*lifetime*_*)* *=* 0.028. The measure of inaccuracy was the absolute log percentage deviation of the estimated probabilities from the true probabilities. The measure of incoherence was the absolute log percentage deviation between the ratio of *P(C|+)* and *P(+|C)* and 0.5, as this ratio should always equal 0.5 based on the information provided to participants. Berg et al. (2016) reported a negative but non-significant correlation between coherence and accuracy (see Table 1). Simulating this experiment many times with the Bayesian sampler, produced an underlying positive correlation of *r =* 0.086, but using a significance level of *0.05*, the chance of the experiment producing a significant correlation was only 21%.

**Table 1:** Empirical and simulated relationship between accuracy and coherence of Wright et al. (1994) and Berg et al. (2016).

Paper	Incoherence measures	Data	Simulations		
		Empirical correlation coefficients between accuracy and coherence	Simulated correlation coefficients from the Bayesian sampler	Power analysis using the Bayesian sampler *P* (significance)	Simulated correlation coefficients using random responses
Wright et al. (1994)	Union discrepancy	0.22	0.551	93.60%	0.191
	Disjunction discrepancy	0.39	0.422	74.60%	0.000
	Intersection discrepancy	0.39	0.519	88.85%	0.236
Berg et al. (2016)	Absolute log percentage deviation	−0.06	0.086	21.04%	0.003

*Note*. Significance level was set at *p* *<* *0.05*. Random responses were probability estimates randomly drawn from a uniform distribution *U[0,1]*.

We also modelled the experiment of Wright et al. (1994), which asked 35 university students to forecast outcomes of quarter-finals, semi-finals, and final in 1989 Snooker World Championship. Accuracy was measured in a number of ways, but we focus on the simplest measure: the number of correct predictions. Coherence was measured three ways: the disagreement of union, intersection, and disjunction judgments from matched combinations of simpler judgments. Wright et al. (1994) found positive relationships between coherence and accuracy for two of these three measures of incoherence. A simulation of the Bayesian sampler was conducted using the parameters for the poker experts, as we also used for the Berg et al. (2016) study. The true probabilities of each player winning a match were calculated from the most recent Elo ranking of snooker players before the 1989 World Championship (Flanagan, 1989). The simulated correlations were strong and the design was high-powered, however, for two of the three measures of incoherence these correlations were confounded: they showed positive correlations between coherence and accuracy even when each simulated response was randomly drawn from a uniform distribution (see Table 1). Only the disjunction discrepancy measure did not show a positive correlation with accuracy when using random simulated responses, and here the Bayesian sampler also predicted a strong positive correlation. This is an important result to highlight as it shows that a correlation between coherence and accuracy can be found for judgments of complex real-world probabilities, and not just when the possibilities can be enumerated as in our “small world” poker task.

## 4 Discussion

We found a positive relationship between the coherence and accuracy of probability estimates both between groups with differing levels of expertise and across individuals within each group. Poker experts were both more coherent and more accurate than novices and amateurs. Within each of the expertise groups, there was also a positive correlation between the coherence and accuracy across individuals. Using secondary measures of extension error counts and degrees of inconsistency between matching questions, the same between-group results and the same within-groups result for poker experts were found. We found these results with coherence and accuracy assessed on different sets of judgments, ensuring that these results were not confounded by the formal link between coherence and accuracy.

Moreover, we were able to reconcile our results with those of previous studies using a computational model. A good match to our data was found using the Bayesian sampler model assuming that participants estimate probabilities based on mental samples, but with the number of samples differing between individuals. Using the same parameters, we predicted positive relationships between coherence and accuracy in past work but showed that at times low experimental power made the positive relationship difficult to detect. Thus, the model reconciled our results, which were based on stimuli with simple objective probabilities, with these other studies, which used ambiguous real-world probabilities, suggesting that the finding is a general one. The successful application of a mental sampling model to both types of judgments has precedence in other work (Costello, Watts, & Fisher, 2018; Sanborn and Chater, 2016; Griffiths, Vul and Sanborn, 2012).

But what links coherence and accuracy? The greater accuracy and coherence of poker experts suggests that the relationship is driven by expertise: by playing poker individuals develop more accurate and coherent strategies for judging card probabilities. Indeed, there is much evidence that people adapt their strategies to task demands in laboratory studies (Lieder & Griffiths, 2017; Rieskamp & Otto, 2006). However, aspects of our data argue against the hypothesis that it is driven by expertise. First, the correlation between coherence and accuracy was found across poker novices who should all lack expertise. Second, when we remove the effect of individual differences in the previous year's poker experiences or the effect of individual differences in the GFM, the correlations between coherence and accuracy do not significantly change. Third, and most persuasively, we found no differences in coherence or accuracy between the Card and Ball tasks, even for individuals who believed the two tasks were fundamentally different. This is a surprise as expertise is often brittle and tends to transfer only between tasks perceived to be similar (Lewandowsky, Little, & Kalish, 2007).

If it is not expertise, one alternative is that higher cognitive capacity or motivation leads some individuals to be generally both more coherent and more accurate. One piece of evidence in favour of this hypothesis is that superforecasters, individuals who have a repeatedly demonstrated a high-level accuracy to predict future events, were found to be more coherent on standard heuristics and biases questions (e.g., Mellers, Baker, Chen, Mandel, & Data, 2017). While not a study of whether coherence and accuracy correlate on the same type of judgments, it is suggestive: superforecasters have no special expertise in the domains in which they make excellent predictions but do have above-average intelligence scores and are more highly motivated than less accurate forecasters (Mellers et al., 2017). Coherence is also generally greater for participants with higher intelligence (Stanovich & West, 2000) and our main incoherence measure in particular is associated with cognitive capacity (Dougherty & Hunter, 2003). Indeed, differences in cognitive capacity or motivation seems most congruent with the assumption in our computational model that there are individual differences in the number of samples drawn. The effect of expertise increasing accuracy but not coherence (e.g., Reyna & Lloyd, 2006) can be modelled in a straightforward way: if expertise causes the underlying probabilities to converge to the correct values without increasing the number of samples drawn, then all else being equal, expertise will improve accuracy but not coherence.

There remains much work to be done both to establish the link in more domains and to directly show that capacity and motivation drive the link between coherence and accuracy. In particular, it will be important to investigate whether this result holds in domains with unmeasurable uncertainty and for judgments and decisions beyond probability estimates. But we can speculate that cognitive capacity or motivation influences the fidelity of approximate Bayesian inference through the number of samples, and this underlies the general link between coherence and accuracy. The work comparing normal adults with superforecasters, whose accuracy is evaluated in domains of unmeasurable uncertainty (Mellers et al., 2017), suggests that the relationship can hold for these domains and that it is driven by group differences in cognitive capacity, but closer comparisons that relate individual cognitive capacity to coherence and accuracy still need to be done. And there are many complications that could weaken the correlation between coherence and accuracy in cases with unmeasurable uncertainty even if individuals rationally update their priors, such as individual differences in the available information sources (Feldman, 2017) or in how people interpret environmental evidence (e.g., Gershman, 2019; Jern, Chang, & Kemp, 2014). Briefly considering judgments and decisions beyond probability estimates, while probability judgments are often hypothesised to be closely tied to confidence and decisions (Kepecs & Mainen, 2012; Pleskac & Busemeyer, 2010) coherence for different responses are often evaluated in different ways (e.g., by assessing choice transitivity) so the link needs to be assessed both theoretically and empirically for these measures. Overall, we anticipate that in some tasks this link will be strong, highlighting the importance of Bayesian rationality, while in other tasks it will be weak, highlighting the importance of ecological rationality.

## 5 Conclusions

An important yet largely neglected question is whether there exists a link between the two major rival theories of rationality: Bayesian rationality (emphasizing coherent beliefs) and ecological rationality (emphasizing correspondence with the world, in which accurate beliefs play a role). The few empirical studies on this topic have shown no link, but here we performed two high-power and unconfounded experiments on judgments of the probability of poker hands. We found a positive relationship between the coherence and accuracy of probability judgments both between groups with differing levels of poker expertise and across individuals within each group. Besides finding strong empirical support of a link between Bayesian and ecological rationality, we also explained both our results and past null results using an approximate Bayesian model, suggesting a way to reconcile these distinct views of rationality.

## Credit Author Statement

J.Q.Z, P.W.S·N., N.C., and A.N·S designed the research; J.Q.Z and P.W.S.N collected data; J.Q.Z analyzed data and performed simulations; and all authors wrote the paper.

## Appendix A Supplementary data

Supplementary material. Image 1

## Footnotes

1 It should be noted that research in the field of ecological rationality does not necessarily discuss ‘accuracy’ per se. Rather, correspondence with the world is typically emphasized, in terms of “relations between behavior and the environment, using measures such as how healthy, rich, successful in school, happy in marriage, or accurate in judgments people are, and ultimately how successful an organism is at survival and reproduction” (Arkes et al., 2016, p. 21), of which accuracy is only one aspect. Additionally, the concept of accuracy in terms of deviation from an objectively ‘true’ probability is seldom discussed in ecological rationality, which concerns itself more with ‘large worlds’ in which probabilities are typically unknown and unmeasurable. This is a common element with the Bayesian perspective, which emphasizes the subjective nature of probability and generally concludes that probabilities only exist in terms of degrees of belief, and that these beliefs should be model-based inferences using environmental frequencies, and so should not perfectly correspond to environmental frequencies themselves but instead should often be smoother and more entropic than those frequencies (Feldman, 2013, Feldman, 2017). Nevertheless, the fact that accuracy cannot always be measured in terms of deviation from an objectively ‘true’ number does not mean that accuracy is meaningless under these circumstances; a probability judgment that does not cohere with the information at hand or that produces a prediction that is explicitly contradicted by subsequent outcomes is arguably an inaccurate judgment.

2 Our participants included the player famous for the biggest win in a day of online poker: $4.2 million (Brian Hastings).

3 The preregistration of Experiment 1 is available at https://osf.io/8scg4

4 We are testing differences between groups using both *t*-tests and Bayesian t-tests. *p* values less than 0.05 are viewed as significant for frequentist *t*-tests. Bayes factor for the alternative hypothesis (i.e., *BF*_*10*_) greater than 3 are often considered as substantial evidence in favour of the alternative, whereas less than 1/3 are often considered as substantial evidence in favour of the null. The Bayes factors throughout this paper were computed using a Jeffrey-Zeller-Siow prior with the scale on effect size equals the default value of 0.707 (Rouder, Speckman, Sun, Morey, & Iverson, 2009).

5 The preregistration of Experiment 2 is available at https://osf.io/vxch4

## References

[bb0005] Adam M.B., Reyna V.F. (2005). Coherence and correspondence criteria for rationality: Experts’ estimation of risks of sexually transmitted infections. Journal of Behavioral Decision Making.

[bb0010] Arkes H.R., Gigerenzer G., Hertwig R. (2016). How bad is incoherence?. Decision.

[bb0015] Berg N., Biele G., Gigerenzer G. (2016). Consistent Bayesians are no more accurate than non-Bayesians: Economists surveyed about PSA. Review of Behavioral Economics.

[bb0020] Costello F., Watts P., Fisher C. (2018). Surprising rationality in probability judgment: Assessing two competing models. Cognition.

[bb3015] Dougherty M.R., Hunter J. (2003). Probability judgment and subadditivity: The role of working memory capacity and constraining retrieval. Memory & Cognition.

[bb0025] Feldman J. (2013). Tuning your priors to the world. Topics in Cognitive Science.

[bb0030] Feldman J. (2017). What are the “true” statistics of the environment?. Cognitive Science.

[bb0035] Flanagan S. (1989). Professional snooker Elo ratings Anglian British open. The Professional Snooker Elo Ratings..

[bb0040] Gershman S.J. (2019). How to never be wrong. Psychonomic Bulletin & Review.

[bb0045] Gigerenzer G. (2019). Axiomatic rationality and ecological rationality. Synthese.

[bb3005] Gigerenzer G., Hoffrage U. (1995). How to improve Bayesian reasoning without instruction: Frequency formats. Psychological Review.

[bb0050] Griffiths T.L., Vul E., Sanborn A.N. (2012). Bridging levels of analysis for probabilistic models of cognition. Current Directions in Psychological Science.

[bb0055] Hammond K.R. (2000). Judgment and Decision Making*:* An Interdisciplinary Reader.

[bb0060] Harnad S. (1987).

[bb0065] Jern A., Chang K.M.K., Kemp C. (2014). Belief polarization is not always irrational. Psychological Review.

[bb0070] Joyce J.M. (1998). A nonpragmatic vindication of probabilism. Philosophy of Science.

[bb0075] Kahneman D., Tversky A. (1973). On the psychology of prediction. Psychological Review.

[bb0080] Kepecs A., Mainen Z.F. (2012). A computational framework for the study of confidence in humans and animals. Philosophical Transactions of the Royal Society B: Biological Sciences.

[bb0085] Leitgeb H., Pettigrew R. (2010). An objective justification of Bayesianism I: Measuring inaccuracy. Philosophy of Science.

[bb0090] Lewandowsky S., Little D., Kalish M.L. (2007). Handbook of Applied Cognition.

[bb0095] Lieder F., Griffiths T.L. (2017). Strategy selection as rational metareasoning. Psychological Review.

[bb0100] Mellers B.A., Baker J.D., Chen E., Mandel D.R., Data P.E.T. (2017). How generalizable is good judgment? A multi-task, multi-benchmark study. Judgment and Decision making.

[bb0105] Newall P. (2013).

[bb0110] Pleskac T.J., Busemeyer J.R. (2010). Two-stage dynamic signal detection: A theory of choice, decision time, and confidence. Psychological Review.

[bb0115] Redelmeier D.A., Koehler D.J., Liberman V., Tversky A. (1995). Probability judgment in medicine: Discounting unspecified possibilities. Medical Decision Making.

[bb0120] Reyna V.F., Lloyd F.J. (2006). Physician decision making and cardiac risk: Effects of knowledge, risk perception, risk tolerance, and fuzzy processing. Journal of Experimental Psychology: Applied.

[bb0125] Rieskamp J., Otto P.E. (2006). SSL: A theory of how people learn to select strategies. Journal of Experimental Psychology: General.

[bb3000] Rouder J.N., Speckman P.L., Sun D., Morey R.D., Iverson G. (2009). Bayesian t tests for accepting and rejecting the null hypothesis. Psychonomic Bulletin & Review.

[bb0135] Sanborn A.N., Chater N. (2016). Bayesian brains without probabilities. Trends in Cognitive Sciences.

[bb0140] Shiffrin R.M., Schneider W. (1977). Controlled and automatic human information processing: II. Perceptual learning, automatic attending and a general theory. Psychological Review.

[bb0145] Stanovich K.E., West R.F. (2000). Individual differences in reasoning: Implications for the rationality debate?. Behavioral and Brain Sciences.

[bb3010] St. Germain J., Tenenbaum G. (2011). Decision-making and thought processes among poker players. High Ability Studies.

[bb0150] Tversky A., Fox C.R. (1995). Weighing risk and uncertainty. Psychological Review.

[bb0155] Tversky A., Kahneman D. (1974). Judgment under uncertainty: Heuristics and biases. Science.

[bb0160] Tversky A., Kahneman D. (1983). Extensional versus intuitive reasoning: The conjunction fallacy in probability judgment. Psychological Review.

[bb0165] Van Rooij I. (2008). The tractable cognition thesis. Cognitive Science.

[bb0170] Van Rooij I., Wareham T. (2012). Intractability and approximation of optimization theories of cognition. Journal of Mathematical Psychology.

[bb0175] Williams R.J. (2003).

[bb0180] Wright G., Rowe G., Bolger F., Gammack J. (1994). Coherence, calibration, and expertise in judgmental probability forecasting. Organizational Behavior and Human Decision Processes.

[bb0185] Zhu J.Q., Sanborn A.N., Chater N. (2020). The Bayesian sampler: Generic Bayesian inference causes incoherence in human probability judgments. Psychological Review.

