# Supplementary material for: Clarifying the relationship between coherence and accuracy in probability judgments
Source: Cognition. 2022 Jun;223:105022. doi: 10.1016/j.cognition.2022.105022 (PMC8987733; doi:10.1016/j.cognition.2022.105022)
Supplement: Supplementary file 1 — Supplementary material. [file mmc1.docx]

**Supplementary Materials**

Experimental data is available from https://osf.io/cdvkn/

**Appendix A**

**Dutch Book Argument and the Additivity Axiom**

For a football match between England and Germany, a gambler can judge an English win with probability *P_1_*, a German win with probability *P_2_*, and a draw with probability *P_3_*.

Table A1.

*An expected-value maximising gambler’s beliefs and betting behaviours.*

| Bets | Degree of belief in outcomes | Is willing to buy when | Is willing to sell when |
| --- | --- | --- | --- |
| £1 if England win | $P_{1}$ | $Q_{1}\leq P_{1}$ | $Q_{1}\geq P_{1}$ |
| £1 if Germany win | $P_{2}$ | $Q_{2}\leq P_{2}$ | $Q_{2}\geq P_{2}$ |
| £1 if draw | $P_{3}$ | $Q_{3}\leq P_{3}$ | $Q_{3}\geq P_{3}$ |

For an £1 bet on any event, a gambler, who estimates the event would happen with probability $p$, would see the bet has the expected value of $£1\times p$. If the gambler maximises his expected value, then he is willing to buy the bet at a price smaller or equal to $£p$. Similarly, he is also willing to sell the same bet to whoever can purchase it from him at a price greater or equal to $£p$.

When this gambler’s belief has violated the additivity axiom, for instance, $P_{1}+P_{2}+P_{3}>1$_,_ a bookie can sell the three bets to the gambler at prices as long as $Q_{1}\leq P_{1}$, $Q_{2}\leq P_{2}$, and $Q_{3}\leq P_{3}$. Regardless of eventual outcomes, the bookie only has to pay the gambler for £1. When $1<Q_{1}+Q_{2}+Q_{3}{\leq P}_{1}+P_{2}+P_{3}$, the bookie can take a sure profit of $Q_{1}+Q_{2}+Q_{3}-£1$ from the gambler where the maximal possible profit is $P_{1}+P_{2}+P_{3}-£1$.

Similarly, if the gambler’s probability estimates of three events add up less than 1, a bookie can buy the three bets from the gambler at prices whenever $Q_{1}\geq P_{1}$, $Q_{2}\geq P_{2}$, and $Q_{3}\geq P_{3}$. Regardless of eventual outcomes, the bookie can get £1 from the gambler. When $P_{1}+P_{2}+P_{3}{\leq Q}_{1}+Q_{2}+Q_{3}<1$, the bookie again obtains a guaranteed profit of ${1-(Q}_{1}+Q_{2}+Q_{3})$ from the gambler where the maximal possible profit is ${£1-(P}_{1}+P_{2}+P_{3})$.

Combined both sub-additive and super-additive beliefs in this example, the maximum sure loss for an expected-value maximiser will be the absolute difference of the sum of his beliefs and 1. This is also our incoherence measure used in the main text, which, in this end, should serve as the upper bound on the exploitation of the incoherent beliefs.

**Appendix B**

**Questionnaire Design**

Here we report the 18 probability judgment queries used in both experiments (see Table B1 and B2). Participants were provided a maximum of 60 seconds to complete each of the 18 probability queries. In addition, we measured participants’ Recent Poker Experiences (RPE) with 3 questions (see Table B3), adapted from Rockloff (2012). For these playtime-related questions, participants were asked to choose a category that best describe the frequency and length of their poker playing sessions. The variable RPE was then calculated based on the median frequency and length of playing time of each categorical response. The questionnaire also included the Gambling Fallacies Measure that asks about general knowledge in probability theory and gambling (see Table B4). Finally, participants were asked whether they noticed a difference between the Card and Ball tasks with the following question: “While you were doing the 3-card task and the 3-ball task, how similar did you think the two tasks were? (a) For every question in the 3-card task, there was a question in the 3-ball task that had exactly the same answer. (b) The questions and answers in the two tasks were not equivalent, but for every ball in the urn there was an equivalent card in the deck. (c) The 3-card task differed in important ways from the 3-ball task”. We considered participants choosing (c) as seeing the two tasks distinct.

Table B1.

*Detailed descriptions and examples for the card task.*

| Card task | Question (For 1000 dealt three-card combination) | Example |
| --- | --- | --- |
| 3-of-a-kind | How many will have three cards of the same rank? | 7♥ 7♣ 7♦, Q♠ Q♥ Q♣ |
| Paired | How many will have two cards of the same rank? | 5♦ 8♠ 5♥, 9♣ 2♠ 2♣ |
| No pair | How many will have three cards of different ranks? | 3♦ 8♦ K♣, J♠ A♠ 10♥ |
| 3-of-a-suit | How many will have three cards of the same suit? | 3♥ 6♥ 10♥, K♣ 2♣ 5♣ |
| 2-of-a-suit | How many will have two cards of the same suit? | 4♦ 9♦ Q♠, J♦ 8♥ 7♥ |
| 3 different suits | How many will have three cards of different suits? | 10♦ 4♣ 10♠, 5♣ J♥ A♠ |
| 3-straight | How many will have three cards to a straight? | 3♣ 2♥ A♦, K♣ A♠ Q♠ |
| 2-straight | How many will have two cards to a straight? | 7♦ 8♠ J♥, A♣ K♠ 7♥ |
| No straight | How many will have no cards to a straight? | 8♣ A♦ 10♣, 2♥ 7♣ 10♥ |

Table B2.

*Detailed descriptions and examples for the ball task.*

| Ball task | Question (For 1000 three-ball draws from the urn) | Example (b=blue, g=green, r=red, y=yellow, M=magic ball) |
| --- | --- | --- |
| 3-of-a-number | How many will have three balls of the same number? | 5b 5g 5r, 11y 11b 11g |
| Paired | How many will have two balls of the same number? | 8g 7g 8b, 3b 6y 6r |
| No pair | How many will have three balls of different numbers? | 4r 5r 9g, 11b Mg 3y |
| 3-of-a-colour | How many will have three balls of the same colour? | 2y 5y My, 11b 5b 12b |
| 2-of-a-colour | How many will have two balls of the same colour? | 6g 9r 2g, 4r 12r 4b |
| 3 different colours | How many will have three balls of different colours? | 3r 3b 8y, 6g 2y Mr |
| 3-straight | How many will have the numbers of the three balls next to one another? | My 1g 2b, 11g 12r Mr |
| 2-straight | How many will have the numbers of two balls next to each other? | 7g 6y 3b, 3b 12b Mr |
| No straight | How many will have the numbers of the three balls not next to one another? | 2b 9b 4r, 7b 10y 1g |

Table B3.

*Detailed descriptions of poker playing time.*

| Questions | Options |
| --- | --- |
| How often did you play poker in the past 12 months? | 1. I have never played poker or I have not played poker at all in the past 12 months 2. Monthly or less 3. 2 to 4 times a month 4. 2 to 3 times a week 5. 4 to 5 times a week 6. 6 or more times a week |
| How much time did you spend playing poker on a typical day in which you played poker in the past 12 months? | 1. Less than 30 min 2. More than 30 min but less than 1 hour 3. More than 1 hour but less than 2 hour 4. More than 2 hour but less than 3 hour 5. More than 3 hour |
| How often did you spend time more than 2 hours playing poker (on a single occasion) in the past 12 months? | 1. Never 2. Less than monthly 3. Monthly 4. Weekly 5. Daily or almost daily |

Table B4.

*Detailed descriptions of the Gambling Fallacies Measure* (Williams, 2003)

| Questions | Options (correct answers in bold) |
| --- | --- |
| Which of the following set of lottery numbers has the greatest probability of being selected as the winning com- bination? | 1. 1,2,3,4,5,6 2. 8, 18, 3, 55, 32, 28 3. **each of the above have an equal probability of being selected** |
| Which gives you the best chance of winning the jackpot on a slot machine? | 1. Playing a slot machine that has not had a jackpot in over a month. 2. Playing a slot machine that had a jackpot an hour ago. 3. **Your chances of winning the jackpot are the same on both machines.** |
| How lucky are you? If 10 people’s names were put into a hat and one name drawn for a prize, how likely is it that *your name* would be chosen? | 1. **About the same likelihood as everyone else** 2. Less likely than other people 3. More likely than other people |
| If you were to buy a lottery ticket, which would be the best place to buy it from? | 1. a place that has sold many previous winning tickets 2. a place that has sold few previous winning tickets 3. **one place is as good as another** |
| A positive attitude or doing good deeds increases your likelihood of winning money when gambling. | 1. **Disagree** 2. Agree |
| A gambler goes to the casino and wins 75% of the time. How many times has he or she likely gone to the casino? | 1. **4 times** 2. 100 times 3. It is just as likely that he has gone either 4 or 100 times |
| You go to a casino with $100 hoping to double your money. Which strategy gives you the best chance of doing this? | 1. **Betting all your money on a single bet** 2. Betting small amounts of money on several different bets 3. Either strategy gives you an equal chance of doubling your money. |
| Which game can you consistently win money at if you use the right strategy? | 1. Slot machines 2. Roulette 3. Bingo (Baccarat for Asian populations) 4. **None of the above** |
| Your chances of winning a lottery are better if you are able to choose your own numbers. | 1. **disagree** 2. agree |
| You have flipped a coin and correctly guessed ‘heads’ 5 times in a row. What are the odds that heads will come up on the next flip. Would you say | 1. **50%** 2. more than 50% 3. or less than 50% |

**Appendix C**

**Detailed Correlation Analyses between Coherence and Accuracy**

In this section, we analyse the pair-wise correlation between coherence and accuracy at the subtask level.

*Figure C1.* Detailed results of correlation between inaccuracy and incoherence for poker novices of Experiment 1. Thirty-one out of 36 correlation coefficients are significantly greater than zero (significance level *p<.05*).

*Figure C2.* Detailed results of correlation between inaccuracy and incoherence for poker amateurs of Experiment 1. Twenty-two out of 36 correlation coefficients are significantly greater than zero (significance level *p<.05*).

*Figure C3.* Detailed results of correlation between inaccuracy and incoherence for poker experts of Experiment 2. Thirty-three out of 36 correlation coefficients are significantly greater than zero (significance level *p<.05*).

**Appendix D**

**Correlation between Inaccuracy and Extension Errors**

In this section, we consider a different incoherence measure, which counts the number of times a participant has violated the extension rule of probability theory. Consider the Card task for instance, we have four pairs of probability queries that must obey the extension rule: (a) 3-of-a-kind must also be 3-different-suits, (b) 3-of-a-kind must also have no straight, (c) 3-straight must also has no pairs, and (d) 3-of-a-suit must also have no pair. Therefore, the number of times the extension rule was violated is an integer between 0 and 4. This also leaves three remaining probability queries that do not have any extensional relationship with other queries: paired, 2-of-a-suit, and 2-straight, which we can use to compute an unconfounded measure of inaccuracy. We then compute the inaccuracy as the sum of absolute difference between people’s estimate and the true probability for these three queries. An equivalent measure can be derived for the Ball task. As shown in Figure D1, positive correlations between inaccuracy and the new incoherence measure are found for all poker groups across both Card and Ball tasks. It is, however, only significantly correlated for the poker experts.

*Figure D1*. Correlation analyses between inaccuracy and number of extension errors for both Card and Ball tasks. Positive correlations were found in all poker groups (left panel: novices, middle panel: amateurs, right panel: experts), but such correlations are only significant for poker experts.

**Appendix E**

**Correlation between Inaccuracy and Inconsistency in the Card and Ball task**

Here we consider a further incoherence measure based on the inconsistency in the matching questions between the Card and Ball task. Recall that, as shown in Figure 1, each question in the Card task shares a same answer with its corresponding one in the Ball task. For instance, 3-of-a-kind from a deck of poker is equivalent to 3-of-a-number from the urn. Given that, we calculate the incoherence measure as absolute difference between the two matching questions within a subtask, and the inaccuracy measure as the absolute difference between the probability estimates and true probabilities for the remaining questions. We also restricted the correlational analysis for participants who reported that the Ball and Card tasks were not different. As shown in Figure E1, E2, and E3, the correlations are mostly positive, except for 4 pairs of coherence and accuracy measures in the poker amateur group.

*Figure E1.* Detailed results of correlation between inaccuracy and incoherence for poker novices of Experiment 1. Three out of 9 correlation coefficients are significantly greater than zero (significance level *p<.05*).

*Figure E2.* Detailed results of correlation between inaccuracy and incoherence for poker amateurs of Experiment 1. None of the correlation coefficients is significantly greater than zero (significance level *p<.05*).

*Figure E3.* Detailed results of correlation between inaccuracy and incoherence for poker experts of Experiment 1. All correlation coefficients are significantly greater than zero (significance level *p<.05*).

**Appendix F**

**The Effects of Recent Poker Experiences and General Gambling Knowledge**

The positive correlations between coherence and accuracy may not solely be driven by the cognitive ability or motivation in approximating true probabilities. In this section, we will conduct further tests on whether Recent Poker Experiences (RPE) and gambling knowledge can also influence the correlation between coherence and accuracy. The RPE was approximated using 3 multiple-choice questions (see Appendix B for details) and the gambling knowledge was tested using the Gambling Fallacies Measure (GFM) developed by Williams (2003). The poker experts spent significantly more times on playing poker than both poker novices and amateurs (*Welch’s t(191.23)=12.69, p<0.01, BF_10_>100*), and also scored higher in gambling knowledge (*Welch’s t(106.35)=10.28, p<0.01, BF_10_>100*).

We performed two partial correlations between coherence and accuracy while controlling for the RPE (Table F1) and GFM (Table F2). Both RPE and GFM had small and significant influence on the original correlation coefficients between coherence and accuracy.

Table F1.

*Bootstrapped results of partial correlation between coherence and accuracy, controlling for recent poker experiences.*

| Group | Mean partial correlation | 95% CI |
| --- | --- | --- |
| Poker novices *(N=45)* | 0.40 | [0.25, 0.54] |
| Poker amateurs *(N=37)* | 0.32 | [0.13, 0.50] |
| Poker experts *(N=186)* | 0.24 | [0.16, 0.32] |

Table F2.

*Bootstrapped results of partial correlation between coherence and accuracy, controlling for gambling fallacies measure.*

| Group | Mean partial correlation | 95% CI |
| --- | --- | --- |
| Poker novices *(N=45)* | 0.38 | [0.24, 0.51] |
| Poker amateurs *(N=37)* | 0.29 | [0.10, 0.47] |
| Poker experts *(N=186)* | 0.24 | [0.16, 0.31] |
